# Lipidomics Profiling of Metformin-Induced Changes in Obesity and Type 2 Diabetes Mellitus: Insights and Biomarker Potential

**DOI:** 10.3390/ph16121717

**Published:** 2023-12-11

**Authors:** Muhammad Mujammami, Shereen M. Aleidi, Adriana Zardini Buzatto, Awad Alshahrani, Reem H. AlMalki, Hicham Benabdelkamel, Mohammed Al Dubayee, Liang Li, Ahmad Aljada, Anas M. Abdel Rahman

**Affiliations:** 1 University Diabetes Center, Medical City, King Saud University, Riyadh 11472, Saudi Arabia; mhmujammami@ksu.edu.sa; 2 Endocrinology and Diabetes Unit, Department of Medicine, College of Medicine, King Saud University, Riyadh 11461, Saudi Arabia; 3 Department of Biopharmaceutics and Clinical Pharmacy, School of Pharmacy, The University of Jordan, Amman 11942, Jordan; s.aleidi@ju.edu.jo; 4 The Metabolomics Innovation Center (TMIC), Edmonton, AB T6G 1C9, Canada; zardinib@ualberta.ca (A.Z.B.); liang.li@ualberta.ca (L.L.); 5 College of Medicine, King Saud Bin Abdulaziz University for Health Sciences, Ministry of National Guard Health Affairs, Riyadh 11426, Saudi Arabia; shahranias@ngha.med.sa (A.A.); aldubayeemo@ngha.med.sa (M.A.D.); 6 Metabolomics Section, Department of Clinical Genomics, Center for Genomics Medicine, King Faisal Specialist Hospital and Research Centre, Riyadh 11211, Saudi Arabia; f1525907@kfshrc.edu.sa; 7 Proteomics Resource Unit, Obesity Research Center, College of Medicine, King Saud University, Riyadh 11461, Saudi Arabia; hbenabdelkamel@ksu.edu.sa; 8 Department of Chemistry, University of Alberta, Edmonton, AB T6G 2G2, Canada; 9 Department of Biochemistry and Molecular Medicine, College of Medicine, Al Faisal University, Riyadh 11461, Saudi Arabia

**Keywords:** lipidomics, high resolution mass spectrometry, metformin, type 2 diabetes mellitus (T2DM), obesity, biomarker

## Abstract

Metformin is the first-line oral medication for treating type 2 diabetes mellitus (T2DM). In the current study, an untargeted lipidomic analytical approach was used to investigate the alterations in the serum lipidome of a cohort of 89 participants, including healthy lean controls and obese diabetic patients, and to examine the alterations associated with metformin administration. A total of 115 lipid molecules were significantly dysregulated (64 up-regulated and 51 down-regulated) in the obese compared to lean controls. However, the levels of 224 lipid molecules were significantly dysregulated (125 up-regulated and 99 down-regulated) in obese diabetic patients compared to the obese group. Metformin administration in obese diabetic patients was associated with significant dysregulation of 54 lipid molecule levels (20 up-regulated and 34 down-regulated). Levels of six molecules belonging to five lipid subclasses were simultaneously dysregulated by the effects of obesity, T2DM, and metformin. These include two putatively annotated triacylglycerols (TGs), one plasmenyl phosphatidylcholine (PC), one phosphatidylglycerol (PGs), one sterol lipid (ST), and one Mannosyl-phosphoinositol ceramide (MIPC). This study provides new insights into our understanding of the lipidomics alterations associated with obesity, T2DM, and metformin and offers a new platform for potential biomarkers for the progression of diabetes and treatment response in obese patients.

## 1. Introduction

Type-2-diabetes mellitus (T2DM) is a chronic metabolic disease defined by abnormally high blood glucose levels (hyperglycemia) due to impaired insulin secretion from pancreatic β-cells or insufficient cell response to insulin, resulting in insulin resistance [1]. T2DM is a very common disease worldwide, and more than 700 million people are predicted to have diabetes by 2045 [2]. The incidence of T2DM is linked to several contributing factors, including genetic factors, obesity, a sedentary lifestyle, insulin resistance, epigenetics, and mitochondrial dysfunction [1].

Obesity is an abnormal and excessive accumulation of body fat to an extent that is associated with a potential health risk [3] (The body mass index (BMI), a measurement based on individual weight and height, is the currently used screening tool for obesity. A BMI greater than or equal to 30 kg/m^2^ indicates the presence of obesity, while a BMI over 25 kg/m^2^ is considered overweight. The prevalence of obesity is now experiencing an alarming increase in the world [3]. It is strongly associated with T2DM, and “diabesity” describes the simultaneous occurrence of obesity and diabetes [4,5]. Almost 80% of the patients with T2DM are usually characterized by being obese with a predominant abdominal fat accumulation, highlighting the link between adiposity and T2DM [4,5]. It is well known that obesity promotes the pathogenesis of T2DM by developing insulin resistance, the hallmark of T2DM [5]. Adipose tissue promotes insulin resistance by activating various inflammatory mechanisms and increasing the release of free fatty acids (FFA), glycerol, hormones, and pro-inflammatory cytokines that stimulate insulin resistance in the peripheral tissues [5,6]. Moreover, the aberrant accumulation and expansion of adipose tissue in cases of obesity would create a microenvironment characterized by hypoactive fatty acid metabolism and fuel cellular stress and pro-inflammatory alterations, which results in increased lipolysis, oxidative stress, mitochondrial dysfunction, hypoxia due to fibrosis, and insufficient angiogenesis. All these obesity-induced changes would also contribute to the development of T2DM [7]. In addition, in cases of obesity, the release of adipokines from the adipose tissue is dysregulated [5]. The most abundant of these adipocytokines is adiponectin, which is found to be negatively associated with obesity and T2DM [8].

This pathogenic association between obesity and T2DM would create a conducive environment for dyslipidemia. Most obese and diabetic patients would have a dysregulated lipid metabolism, clinically manifested as hyperlipidemia, characterized by increased levels of triglycerides and low-density lipoprotein cholesterol (LDL-C) [9]. High levels of LDL-C pose a significant risk for cardiovascular diseases, including ischemic heart disease and stroke, and consequently, a substantial global health burden [10]. Strategies such as preventive measures, early detection biomarkers, and effective lipid-lowering agents are essential to mitigating the hyperlipidemia-associated health, economic, and social impacts [10]. Statins are the cornerstone lipid-lowering agents. However, the current lipid management practices [11] highlight the use of new therapeutic agents such as anti-proprotein convertase subtilisin/kexin type 9 (PCSK9) monoclonal antibodies (PCSK9 inhibitors) and ezetimibe, particularly for patients with high cardiovascular risk, including those who are obese and diabetic [11]. Therefore, understanding the alterations underlying the molecular link between obesity, insulin resistance, T2DM, and lipid profiles is pivotal for disease prevention and effective treatment.

Metformin is the first-line oral medication for the treatment of T2DM. It can be used as a monotherapy in the early stages of the disease or combination with other antidiabetic medications [12]. Metformin has multiple actions to lower blood glucose levels, including enhancing insulin sensitivity in the peripheral tissue by increasing peripheral glucose uptake, reducing intestinal glucose absorption, and inhibiting hepatic gluconeogenesis [13]. Moreover, it affects ATP production pathways, including glycolysis and oxidative phosphorylation [13,14]. Beyond its antidiabetic activity, metformin has documented effects on weight and waist circumference reduction, particularly in obese and overweight patients [15,16]. In addition, metformin has a role in the management of women with polycystic ovary syndrome (PCOS) as an effective agent for inducing ovulation in non-obese women with PCOS [17]. In addition, it has anti-inflammatory properties, a reduction of oxidative stress [18], and anti-aging activity [19]. Moreover, metformin has lipid-modifying effects. Its administration was associated with a reduction in the levels of circulating triglycerides (TGs) and total cholesterol and an increase in the levels of high-density lipoprotein (HDL) [20,21]. In addition, using metformin was associated with significantly lower risks of cardiovascular disease in patients with T2DM [22]. Despite the numerous studies about metformin and its multiple actions, it is still necessary to understand its effects using advanced analytical approaches, particularly in diabetic-obese patients.

Recently, we have investigated the metabolic effects of metformin in T2DM and its impact on obesity and insulin resistance using an untargeted mass spectrometry-based metabolomics approach [23,24]. In addition, we have examined the effects of short-term metformin administration on lipid metabolisms and biochemical pathways in healthy individuals [25,26]. In the current study, we used untargeted lipidomics, a comprehensive, advanced analytical approach, to investigate the changes in the serum lipidome of a cohort of healthy lean and obese individuals and examine alterations in the serum lipidome associated with metformin administration in obese T2DM patients.

## 2. Results

### 2.1. Demographics and the Clinical Characteristics of this Study Population

Table 1 shows the demographics and clinical characteristics of this study population. All this study groups, including non-diabetic control (*n* = 28), non-diabetic obese (*n* = 21), obese diabetic (OT2DM) (*n* = 16), and obese diabetic on metformin (OT2DMMet) (*n* = 24), have a significant difference in age (*p*-value < 0.05). The number of males and females is also not equal between this study groups: there are more females in the obese and the OT2MMet groups, while there are more males in the control and the OT2DM groups (Table 1). Among the recruited controls, there are only 3 subjects with a BMI between 18 and 20 kg/m^2^ (underweight), and this proportion accounted for 10% of the control group. Regarding the medication used, including cholesterol-lowering and antihypertensive agents, only six patients among the OT2DMMet (25%) were taking statins (10–20 mg), and only seven OT2DMMet patients (29%) were taking antihypertensive agents.

### 2.2. Lipid Detection and Data Overview Using Multivariate Analysis

**Figure 1:**
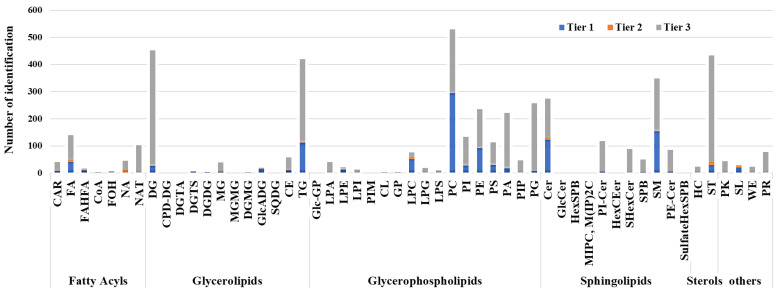
Lipid class distribution for identified features. Compounds identified by tandem-MS with MS/MS score ≥500 and precursor *m*/*z* error ≤5.0 mDa and 20.0 ppm were considered Tier 1; those identified by tandem-MS with MS/MS score <500 and precursor *m*/*z* error ≤5.0 mDa and 20.0 ppm were considered Tier 2; and compounds identified by mass-match with *m*/*z* error ≤5.0 mDa and 20.0 ppm were deemed Tier 3.

A total of 12,877 features (an average of 13,419 ± 920 per sample analysis) were aligned and employed for identification. A three-tier identification approach embedded in NovaMT LipidScreener was employed to annotate lipid features putatively. Among the 12877 unique peaks detected after data processing, 5329 (Supplementary Table S2) were annotated (abbreviations are presented in Supplementary Table S1). We applied a 10-tier filtering and scoring approach based on the characteristics and behavior expected for each lipid class within the employed method and biological samples to select the best identification for isomeric/isobaric species and calculate match scores. The weighted scores from every 10 tiers were combined to calculate a final match score for each annotated feature. In Tier 1, 1168 features were annotated using tandem-MS (MS/MS match score ≥500) and a precursor mass filter (*m*/*z* tolerance of 20.0 ppm and 5.0 mDa). In Tier 2, 89 additional features were annotated using tandem-MS (MS/MS match score <500 and *m*/*z* tolerance of 20.0 ppm and 5.0 mDa). In tier 3, the remaining unannotated features were searched on NovaMT LipidScreener for mass match, and 4072 features were annotated. The annotated lipid classes include fatty acyls, glycerolipids (GL), glycerophospholipids (GPL), sphingolipids, sterols, and others (polyketides, prenols, saccharolipids, and compounds not classified as lipids) (Figure 1).

In addition, out of the 5329 annotated compounds, 885 (16.6%) contained vinyl-ether bonds (plasmalogens), 3507 (65.8%) included polyunsaturated fatty acids (PUFA), 1928 (36.2%) had odd-chain fatty acids (OCFA), and 2561 (48.1%) were annotated with extra oxygen atoms that could indicate oxidation.

**Figure 2:**
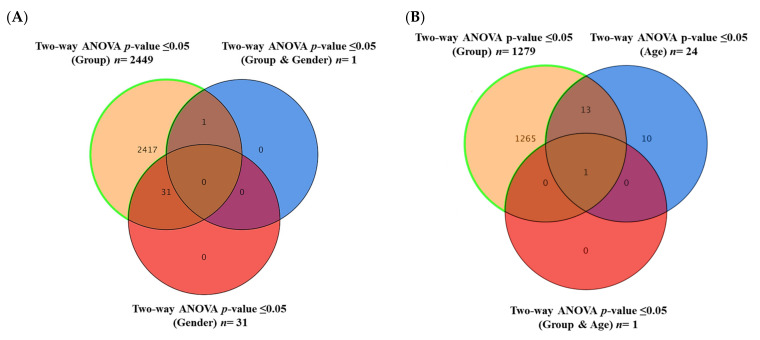
Sequential extraction of gender- and age-independent lipids from the panel of overall detected features (**A**) The Venn diagram shows 2417 lipid molecules are gender-free after performing a two-way ANOVA on 5329 to exclude the gender effect from the detected lipid molecules (groups and gender). (**B**) The Venn diagram shows 1265 lipid molecules that are gender- and age-independent after applying a two-way ANOVA to 2417.

As presented in Section 2.1, there was a significant difference between these study groups in demographic data, including age and gender. These were considered confounding factors; therefore, their effect was extracted from the data set (*n* = 5329) to enhance the validity of the findings. Using Venn diagram analysis and a two-way ANOVA with an FDR corrected *p*-value (FDRp) cut-off = 0.05, the gender (Figure 2A) and age (Figure 2B) independent lipid molecules (*n* = 1265) (Supplementary Table S3) were extracted sequentially from the overall detected features (*n* = 5329) and used throughout the conducted analysis.

**Figure 3:**
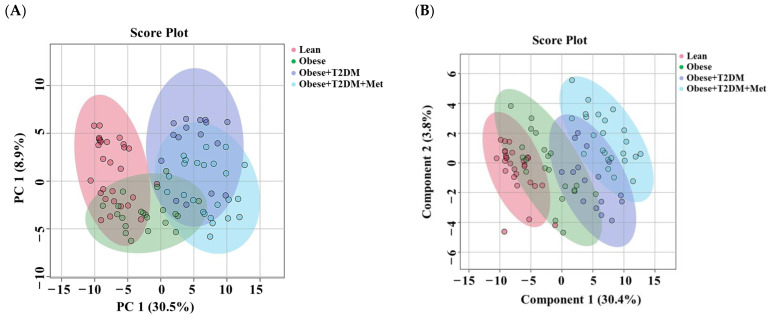
Lipidomic profile of this study group. The lipidomes of serum samples from this study group were evaluated using (**A**) PCA score plots using the class order matters option and (**B**) PLS-DA score plots based on 1265. Control, *n* = 28; obese, *n* = 21; obese with type 2 diabetes mellitus (OT2DM), *n* = 16; and obese with type 2 diabetes mellitus taking metformin (OT2DMMet, *n* = 24).

The age- and gender-independent lipidomics profile (*n* = 1265) of this study group was examined using multivariate analysis. The principal component analysis (PCA) score plot showed an overlap between some of these study groups, with a slight separation between Lean and each of the obese+T2DM (OT2DM) and Obese+T2DM+Met (OT2DMMet) groups (Figure 3A). The supervised analysis using a partial least squares discriminant analysis (PLS-DA) score plot showed a clustering of the diabetics’ group versus non-diabetics but with no apparent separation between the datasets (Figure 3B). However, in both models, the lean control group was separated from the diabetics’ group, which can be observed in the PLS-DA score plots.

### 2.3. Lipidomic Alterations Associated with Obesity in Non-Diabetic Subjects

**Figure 4:**
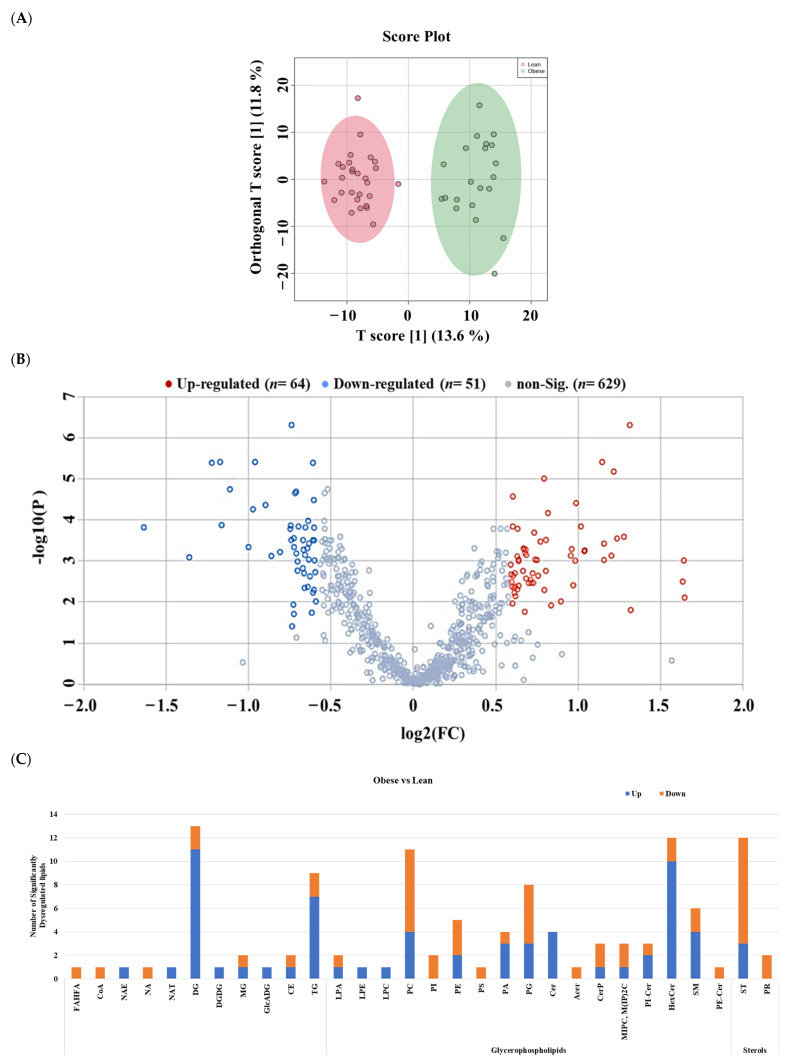
Lipidomic profile of obese versus lean subjects. (**A**) OPLS-DA shows separation between lean and obese groups (R^2^: 0.929; Q^2^: 0.724) based on 1265 (age-gender free lipid molecules). (**B**) The volcano plot revealed 115 out of 1265 dysregulated lipid molecules, where 64 (Red) and 51 (Blue) molecules were up- and down-regulated in obese subjects compared to lean control subjects, respectively (Cut-off: FDR *p*-value ≤ 0.05 and FC 1.5). (age and gender-independent). (**C**) A bar graph shows the distribution of significantly dysregulated lipids between obese and lean (*n* = 115) on lipid subclasses.

The lipidomics alterations among the detected features (*n* = 1265) associated with obesity in the absence of T2DM were examined by comparing the lean controls (*n* = 28) with the obese non-diabetics (*n* =21) group. The supervised analysis using OPLS-DA showed apparently clear group separation and sample clustering between lean and obese subjects (R^2^ = 0.929, Q^2^ = 0.724), indicating a possible effect of obesity on the serum lipidome profile (Figure 4A). Univariate analysis through a volcano plot was performed to investigate the significantly up- and down-regulated lipid molecules between obese and lean groups, where the cut-offs were FDR *p*-value < 0.05 and FC 1.5 (Figure 4B). A total of 115 lipid molecules were significantly dysregulated in the obese group compared to the lean group, where 64 and 51 lipid molecules were up- and down-regulated, respectively (Supplementary Table S4). Diacylglycerols (DGs), Hexosyl ceramides (HexCer), and Triacylglycerols (TGs) were the most upregulated subclass in the obese compared to lean controls (*n* = 11, 10, and 7, respectively). In contrast, Sterol Lipids (ST), Phosphatidylcholines (PCs), and Phosphatidylglycerols (PGs) were mostly downregulated (ST, *n* = 9, PCs, *n* = 7, PGs, *n* = 5, respectively) (Figure 4C).

### 2.4. Lipidomic Alterations Associated with T2DM in Obese Patients

**Figure 5:**
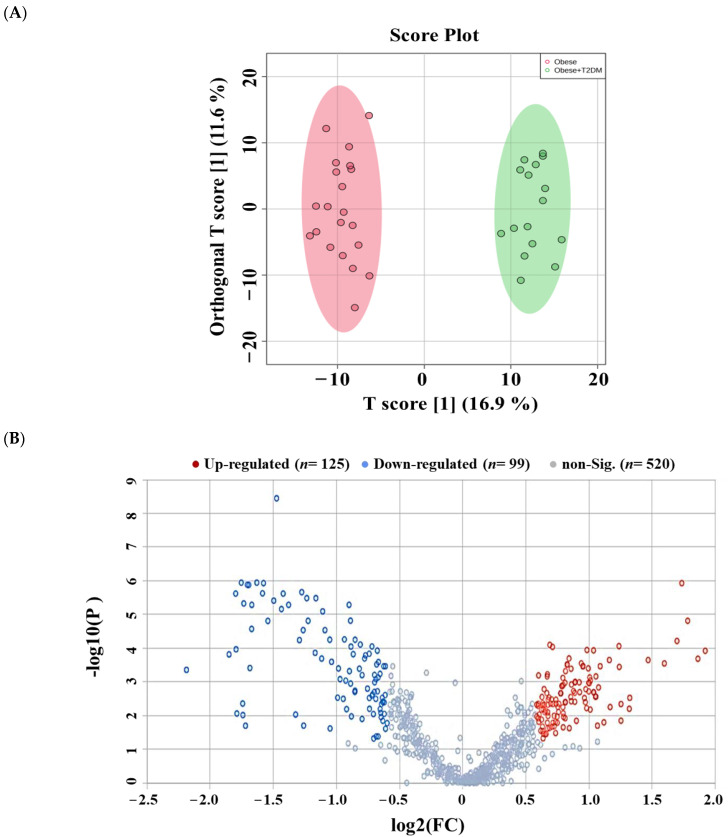

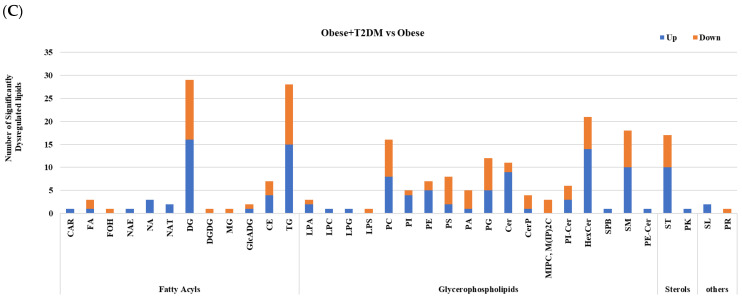
Lipidomic profile of obese diabetics versus obese non-diabetics. (**A**) OPLS-DA clearly separates obese and obese diabetic groups (R^2^: 0.973; Q^2^: 0.822). (**B**) The volcano plot revealed 224 dysregulated lipid molecules, where 125 (Red), and 99 (Blue) lipid molecules were up- and down-regulated in the obese diabetic group compared to the obese group, respectively (Cut-off: FDR *p*-value ≤ 0.05 and FC 1.5). This analysis is based on 1265 lipid molecules (age and gender-independent). (**C**) A bar graph shows the distribution of significantly dysregulated lipids (*n* = 224) on lipid subclasses between the obese diabetic group and the obese group.

The lipidomics changes associated with T2DM in cases of obesity among the detected age- and gender-independent lipids (*n* = 1265) were investigated based on a comparison between obese diabetic patients (*n* = 16) and obese non-diabetics (*n* = 21). An evident separation was noted in the OPLS-DA score plot (R^2^ = 0.973, Q^2^ = 0.822) (Figure 5A), suggesting T2DM significantly affects the serum lipidome of obese individuals.

A volcano plot (Cut-offs FDR *p*-value < 0.05 and FC 1.5) revealed a significant dysregulation in the levels of 224 lipid molecules, where the levels of 125 were up- and 99 down-regulated in obese diabetics compared to the obese group (Figure 5B). The identity of these lipid molecules and their changed levels with fold change and *p*-values are presented in Supplementary Table S5. Moreover, the distribution of the significantly dysregulated molecules (*n* = 224) on lipid subclasses in obese diabetics compared with obese non-diabetics is shown in Figure 5C. Thirty-four lipid subclasses were dysregulated in obese subjects due to T2DM. Among them, TGs and DGs were mostly dysregulated (TGs; *n* = 28, 16 up and 13 downregulated, and DGs; *n* = 29.15 up and 13 downregulated, Figure 5C). In addition, several GLP classes were dysregulated due to T2DM. Among them, PCs (*n* = 16), PGs (*n* = 12), HexCer (*n* = 21), and SM (*n* = 18) were the most dysregulated in obese diabetic subjects (Figure 5C, Table S5).

### 2.5. Lipidomic Alterations Associated with Metformin Administration in Obese Type 2 Diabetic Patients (OT2DM)

**Figure 6:**
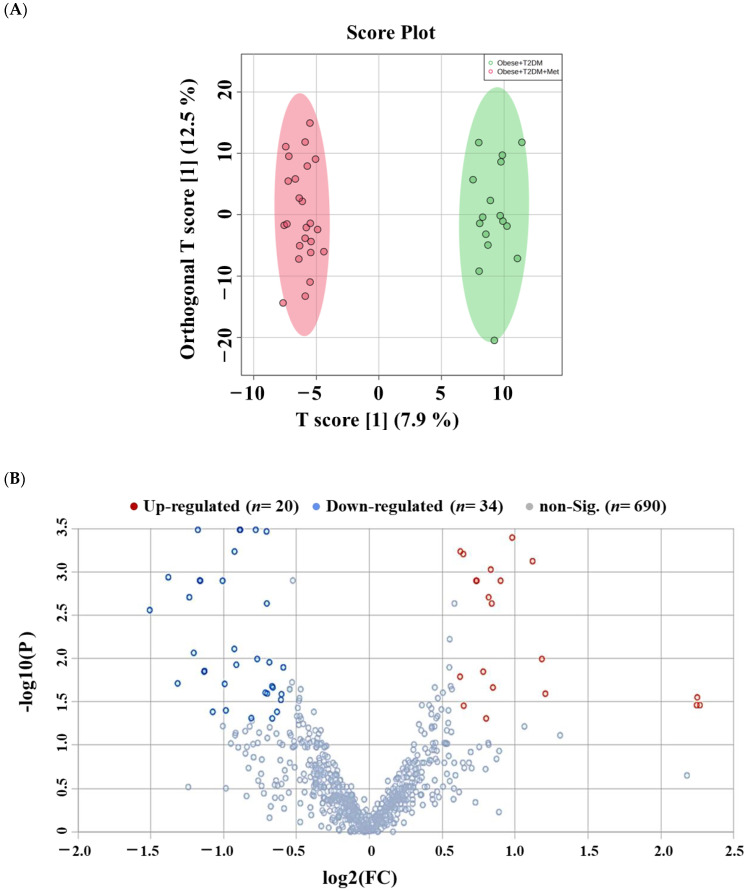

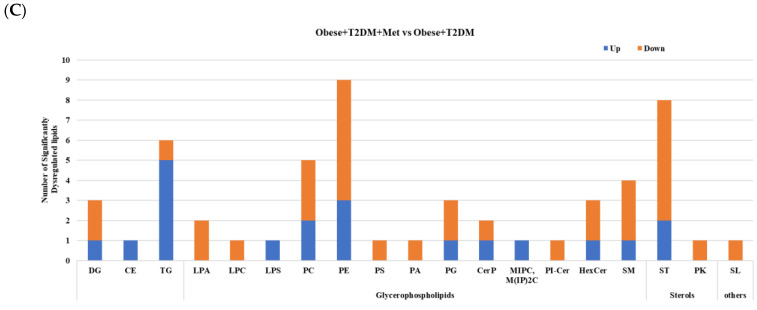
Lipidomic profile of obese diabetics on metformin versus obese diabetics. (**A**) OPLS-DA shows a clear separation between obese diabetics on metformin and obese diabetic groups (R^2^: 0.983; Q^2^: 0.751). (**B**) The volcano plot revealed 54 dysregulated lipid molecules, where 20 (Red) and 34 (Blue) lipid molecules were up- and down-regulated in the (OT2DMMet) vs. (OT2DM) group, respectively (Cut-off: FDR *p*-value ≤ 0.05 and FC 1.5). This analysis is based on 1265 lipid molecules (age and gender-independent). (**C**) A bar graph shows the distribution of significantly altered lipids as an effect of metformin in obese diabetics (*n* = 54).

The effect of metformin administration on the serum lipidome of obese diabetic patients was examined based on a comparison between the OT2DMMet (*n* = 24) and OT2DM (*n* = 16) groups. The OPLS-DA score plot showed an obvious separation and clustering between the compared groups (R^2^ = 0.983, Q^2^ = 0.751, Figure 6A). Considering the age and gender-independent group of lipids (*n* = 1265), volcano plot analysis revealed that 54 lipid molecules were significantly altered when metformin was administered to obese diabetic patients. Among them, 20 and 34 lipid molecules were up- and down-regulated, respectively, in OT2DMMet compared to OT2DM (Cut-off: FDR *p*-value ≤ 0.05 and FC 1.5, Figure 6B). The identity of these dysregulated lipid molecules is presented in Supplementary Table S6. The significantly altered lipid molecules (*n* = 54) were distributed among nineteen lipid subclasses (Figure 6C). Specifically, TGs, PCs, PEs, and ST were the most dysregulated subclasses for the metformin comparison (Figure 6C and Table S6). Interestingly, 83.3% of TGs were mostly up-regulated (5 molecules out of 6), while lipid molecules in other subclasses (60% PCs, 66.6% PEs, and 75% ST) were down-regulated in OT2DMMet compared to OT2DM (Figure 6C and Table S6).

### 2.6. Commonly Altered Lipid Molecules as Effects of Obesity, T2DM, and Metformin Administration

To investigate the altered lipid molecules simultaneously with the presence of obesity, T2DM, and metformin, an overlap between the dysregulated lipid molecules as an effect of obesity, indicated by BMI (BMI panel, *n* = 115), T2DM (T2DM panel, *n* = 224), and metformin administration (Metformin panel, *n* = 54), was carried out using Venn diagram analysis and a moderated *t*-test (*p*-value < 0.05). The results highlighted five lipid subclasses as common and significantly altered between the three panels. The identity of the significantly dysregulated lipid molecules, subclasses, and regulation patterns due to BMI, T2DM, and metformin administration is presented in Table 2.

## 3. Discussion

Metformin is a biguanide oral drug widely used as a first-line medication for the initial treatment of T2DM. It has a role in enhancing insulin sensitization and inhibiting gluconeogenesis [27]. Metformin has been prescribed for other indications, such as PCOS and obesity associated with insulin resistance. Using the LC-MS-based metabolomics approach, we have previously reported significant alterations in the metabolic pattern of long-term metformin administration in diabetic, obese, and lean patients [23]. In addition, it has been shown that metformin affects the metabolome and lipid metabolism after single-dose intake in healthy subjects [25,26]. However, its impact on the lipid metabolism associated with metformin intake in diabetic patients has not been investigated yet. In the current study, the LC-MS-based lipidomics analysis of serum samples from obese non-diabetic and obese diabetic patients taking metformin was conducted to explore the lipidomics profiles and to investigate the lipid molecule alterations associated with metformin administration in obese diabetic patients.

The age- and gender-specific lipid signature in metabolic disorders associated with obesity has been reported [28,29,30]. In our analysis, the effect of confounding factors, including gender and age, were considered and excluded from the dataset. Therefore, the putatively annotated dysregulated lipid molecules were specifically dependent on metformin administration in obese diabetic patients.

The significantly altered lipid molecules simultaneously with the presence of obesity, T2DM, and metformin include triacylglycerols (TGs), plasmenyl phosphatidylcholine (PC), phosphatidylglycerol (PGs), sterol lipid (ST), and Mannosyl-phosphoinositol ceramide (MIPC). MIPCs are a class of lipids strictly related to fungi membranes, where the fungi may be part of the human microbiome, and some sources are pathogenic. These belong to phospholipids, glycolipids, and phosphosphingolipids within the sphingolipids. The main biological functions of these lipid molecules were structural components of the lipid bilayer of cells, metabolic fuels, and signaling molecules. It is well known that regulating lipid metabolism pathways is tightly associated with obesity. Moreover, factors associated with obesity, such as the availability of saturated fatty acids [31] and circulating inflammatory cytokines, selectively activate enzymes that promote lipid synthesis, such as sphingolipids [32]. Therefore, comprehensive lipidomics profiling of obese subjects would provide new insights into our understanding of lipid metabolism in relation to obesity and its associated metabolic diseases, such as T2DM. Our analysis revealed that most of the upregulated lipid molecules affecting obesity/BMI belong to glycerolipids, such as TGs and DGs, and sphingolipids, such as HexCer. It has been reported that obesity is associated with dysregulation in sphingolipid metabolism, particularly ceramides, and insulin resistance in tissues such as adipose tissue, liver, and skeletal muscle [33,34,35]. In addition, our results showed that the levels of GPLs, including PCs, PGs, and ST, were dysregulated in obese subjects. In line with our findings, lipidomic profiling of plasma samples from large cohorts indicated that glycerolipids and sphingolipids had a strong positive association. In contrast, GPLs, including PC species, had a negative association with increasing waist circumference and BMI [29,36].

Clinically, the association between T2DM and dyslipidemia is well established, and dyslipidemia incidence is very common, especially in obese diabetic patients [37]. The clinical components of diabetic dyslipidemia involve an increase in the levels of low-density lipoprotein cholesterol (LDL-C) and triglycerides (TGs) associated with a decrease in the levels of high-density lipoprotein cholesterol (HDL-C) particles [37]. In addition, it is associated with increased levels of other atherogenic lipoproteins, including LDL, very low-density lipoprotein (VLDL), and intermediate-density lipoproteins [38,39]. All the aforementioned lipoproteins, except HDL, contain apoB, a key component of the atherogenic lipoproteins, representing their total number in the circulation [39]. Therefore, considering apoB levels provides more accurate clinical lipidomics for diabetic patients. Several recent studies have examined the lipidomics profiles of diabetic patients versus healthy subjects [40,41]. In addition, a recent systematic review and meta-analysis of prospective cohort studies indicated that glycerolipids (DGs and TGs), lysophosphatidylethanolamines (LPE), and Cer were associated with a higher risk of T2DM [42]. In the current study, lipidomics profiling of obese diabetics versus obese non-diabetics revealed dysregulation of 224 lipid molecules. Consistent with previous lipidomics analysis [35,41], our analysis showed that sphingolipids, including Cer, HexCer, and sphingomyelin (SM), were upregulated in the diabetics’ group. The association between Cer and insulin resistance is evident [43]. Ceramides [11] are bioactive lipids and part of the membrane microdomains, called lipid rafts. They are important in mediating different cell signaling pathways, including insulin signaling [43]. In addition, Cer has been implicated as an antagonist of insulin action by inhibiting Akt/PKB [44]. Thus, the accumulation of Cer could interfere with glucose uptake, indicating a progression of T2DM.

Among the significantly dysregulated lipid subclasses due to obesity and T2DM were TGs and DGs. A central pathway that connects lipid and glucose metabolisms is the glycerolipid/free fatty acid (GL/FFA) cycle [45]. This cycle has an important role in maintaining the balance between lipogenesis and lipolysis via the formation of TGs through the esterification of FFA glucose-derived glycerol, the hydrolysis of TG, and the release of FFA and glycerol, respectively [45]. Insulin is crucial in regulating the key enzymes participating in this cycle. It has an activating effect on the lipoprotein lipase enzyme in the circulation and inhibitory effects on the hormone-sensitive lipase in the adipose tissue. Lipidomic and transcriptomic analyses of nerve tissue biopsies from hyperlipidaemic diabetic patients with peripheral neuropathy revealed an increase in the expression of the diacylglycerol acyltransferase 2 (DGAT2) enzyme, which mediates the committed step in TG synthesis [46]. Therefore, abnormalities and perturbations in the GL/FAA cycle would lead to metabolic diseases, including obesity, insulin resistance, T2DM, and hyperlipidemia [45]. Moreover, animal studies using a diabetic mouse model indicated that diabetes is associated with an accumulation of lipid hydroperoxides and, thus, an increase in oxidative stress [47]. In line with this, most of the detected upregulated lipid molecules were oxidized as an effect of T2DM (Table S5).

Metformin-associated lipidomics alterations have been reported in animal models [48], healthy human subjects [26], and PCOS women [49]. The annotated lipidomics signature due to metformin included alterations in sphingolipid metabolism, GPL metabolism, and specifically a decrease in oxidized lipids [26,48,49], confirming the protective effect of metformin on the oxidative stress status [50].

The effect of metformin administration on the lipidomics profiles of obese diabetic patients was examined in this study. The levels of several lipid molecules were significantly altered by metformin. They were distributed among nineteen lipid subclasses, including DGs, TGs, PCs, PEs, PGs, Hex2Cer, SM, and ST. Interestingly, most TG molecules were up-regulated, while molecules in other lipid subclasses, such as PCs, PEs, and ST were down-regulated in diabetic patients receiving metformin. It has been shown that metformin affects phospholipid metabolism in various ways. One of the underlying anti-diabetic effects of metformin is mediated by the activation of AMP-activated protein kinase (AMPK). This is cellular energy sensor that has a regulatory role in lipid metabolism, including altered synthesis and breakdown of various lipid molecules [51,52]. However, the specific effects of metformin on lipid metabolism and the levels of circulating lipid molecules depend on various factors, including the overall patient’s lipid profile, genetic and environmental factors, the patient’s characteristics, and, on top of these, the presence of metabolic disorders such as obesity and T2DM.

The results revealed common significant lipid subclasses simultaneously dysregulated by obesity, T2DM, and metformin (Table 2).

The pattern of these lipid subclass alterations was up-regulation in obese people, down-regulation in diabetics, and then up-regulation as an effect of metformin, reaffirming the role of metformin in enhancing insulin sensitivity and glucose uptake by cells and in regulating lipid metabolism. Moreover, the different patterns annotated in the regulations of these annotated lipid molecules indicate that each status (obesity, T2DM, metformin) impacts the patients’ lipidomics profiles differently.

Among the common and significantly altered lipid molecules detected were TG species that were upregulated in obese diabetic patients receiving metformin. TGs are circulating lipids transported in the bloodstream via very-low-density lipoprotein (VLDL) and LDL. High levels of TGs are associated mainly with elevated VLDL, an atherogenic lipoprotein that contributes to atherosclerosis development. The effect of metformin on the TGs and their transporting lipoproteins, mainly VLDL, clearance, and metabolism, still needs to be fully understood. Recently, it has been reported that 12-week metformin treatment in obese humans with non-alcoholic fatty liver disease (NAFLD) resulted in a significant decrease in VLDL-TG concentrations [53]. On the other hand, three-month metformin treatment in a randomized, placebo-controlled clinical trial in T2DM patients indicated no change in the VLDL-TGs levels after treatment [54].

This study has some limitations, particularly in characterizing the recruited patients. Given that T2DM is a common metabolic disease among overweight and obese individuals, it was difficult to find and recruit diabetic lean patients to compare them with diabetic obese patients to examine the effect of obesity on the lipidomics profile of diabetic patients. In addition, recruiting healthy controls matched in age with the included diabetic patients was challenging in this study as most T2DM patients are elderly or over 35. Therefore, the sample size of 89 participants would limit the generalizability of this study findings and thus require further validation in a large-scale cohort. In a future study, we are aiming to recruit an independent cohort to validate the potential biomarkers discovered using a targeted approach using tandem mass spectrometry and reference standard materials for absolute quantification.

Despite these limitations, we excluded the effect of confounding factors, including age, in our analysis and carried out multiple comparisons to extract the significant alterations in the annotated lipid molecules. Furthermore, this is a prospective study based on untargeted, comprehensive lipidomics. As such, we recognize the potential for false-positive annotation of lipid species, particularly for the Tier 3 IDs. Follow-up investigations will focus on the confirmation of lipid annotations.

## 4. Materials and Methods

### 4.1. Study Population

A cohort of 89 participants was involved in this study. They were divided into two main groups: (non-diabetics (*n* = 49) and diabetics (*n* = 40)). The non-diabetic group included lean, healthy subjects (control, *n* = 28) and obese subjects (*n* = 21). The diabetics’ group included obese diabetics (OT2DM, *n* =16) and obese diabetics on metformin (500–1500 mg/day) (OT2DMMet, *n* = 24) for at least six months. The participants in this study were recruited from a primary healthcare hospital at King Abdulaziz Medical City (Riyadh, Saudi Arabia). Our recent work [23,24] mentioned recruitment details and inclusion criteria.

### 4.2. Ethics Statement

All procedures performed in this study involving human participants followed the ethical standards of the Declaration of Helsinki and the Universal International Conference on Harmonization-Good Clinical Practice (ICH-GCP) guidelines. This study was reviewed and approved by the Institutional Review Board (IRB) at the King Abdulaziz Medical City Ethics Committee (Protocol # RC12/105). Written informed consent was obtained from all participants before they participated in this study.

### 4.3. Anthropometric Measurements

The body mass index (BMI) for each participant was calculated as body weight (in kilograms) divided by the square of body height (in meters). The BMI was classified into normal (18–24.9 kg/m^2^), overweight (25–29.9 kg/m^2^), obese (30–34.9 kg/m^2^), and morbidly obese (≥35 kg/m^2^). Obese and morbidly obese participants were included in the obese group, while healthy BMI was included in the lean non-obese group. Since the overweight group included a small number of participants (8 subjects-5 healthy subjects and 3 T2DM patients), they were split as “lean” (BMI ≤ 26 kg/m^2^) and "obese" (BMI > 26 kg/m^2^).

### 4.4. Sample Preparation and Liquid Chromatography-Mass Spectrometry (LC-MS) Analysis

Samples were blindly randomized into eleven batches of six to nine for preparation and LC-MS analysis. A pooled mixture containing one aliquot of each sample was prepared for quality control (QC). Each batch of samples was prepared and analyzed along with one experimental replicate of the QC pool to ensure experimental reproducibility and control of potential batch effects. All samples and QC aliquots were prepared with an internal standard mixture composed of 15 deuterated lipids (NovaMT LipidRep Internal Standard Basic Mix for Serum/Plasma, Nova Medical Testing, Inc., Edmonton, AB, Canada), added to the samples immediately after aliquoting to ensure control of all analytical steps. The mixture included different concentrations of [D5]LPC 18:1, [D5]LPE 18:1, [D5]MG 18:1, [D3]FA 16:0, [D3]ST 27:1;O ([D3]Cholesterol), [D5]PG 16:0_18:1, [D5]PS 16:0_18:1, [D5]PA 16:0_18:1, [D5]PC 16:0_18:1, [D5]PE 16:0_18:1, [D3]Cer 16:0;O2/18:1, [D5]DG 16:0_18:1, [D5]TG 16:0_18:1_16:0, and [D3]CE 18:1. The 15 deuterated lipid standards were synthesized in-house. The composition of the mixture was carefully optimized to match the expected intensity of lipids found in human serum. Blank extractions of water instead of the sample were performed before and after all sample extractions to control sources of contamination.

A modified version of the classical Folch liquid-liquid extraction method with dichloromethane and methanol-extracted lipids was used, as previously published [55]. Briefly, aliquots of 6.0 µL of serum samples were vortexed with 6.0 µL of NovaMT LipidRep Internal Standard Basic Mix for Serum/Plasma. Lipids were extracted with 2:1 dichloromethane/methanol, followed by a clean-up step with water (8:4:3 dichloromethane/methanol/water). The mixture was equilibrated at room temperature for 10 min, followed by centrifugation for 10 min at 4 °C and 12,000 rpm. A fraction of the organic layer was evaporated to dryness using a nitrogen blow-down evaporator. The dried residue was re-suspended in 48 µL (8-fold dilution) of 10% of mobile phase B [56] and 90% of mobile phase A [57]. The extracts were immediately transferred to inserts placed inside autosampler vials with PTFE/silicone caps and kept at 4 °C until analysis. All samples were injected between 4 h and 28 h after extraction.

Chromatography conditions for the reversed-phase LC-ESI-QToF-MS sample analysis were MPA-10 mmol/L ammonium formate in 50:40:10 methanol/acetonitrile/water, MPB-10 mmol/L 95:5 2-propanol/water, 250 µL/min, 42 °C, gradient elution (0 min—5% MPB; 10 min—40% MPB; 18.8 min—98% MPB; 20.5 min—98% MPB; 21.2 min—5% MPB) followed by 5 min re-equilibrium, and injection volume of 3.5 µL for positive ionization and 10.0 µL for negative ionization. LC-MS/MS data were acquired for all samples in auto-MS/MS mode using Bruker oToF Control (cycle time of 1.2 s; MS1 acquisition rate of 1.44 Hz; dynamic MS2 acquisition rate between 4.0 and 10.0 Hz; and collision energies between 28.0 and 42.0 eV). Each sample was injected once for positive and once for negative electrospray ionization within *m*/*z* 150 to 1500. Additional LC-MS/MS injections were acquired with the QC pooled mixture using different collision energies (between 5 and 80 eV), injection volumes (2 to 5 µL for positive ionization and 8 to 12 µL for negative ionization), and precursor mass ranges. Each randomized batch of six to nine samples was injected between two injection replicates of the corresponding QC experimental replicate. Multiple replicates of the QC mixture were also prepared and injected before and after all samples to ensure stable and reproducible analysis conditions. The acceptance criteria were to have all the QC samples separated from the other study groups, clustered together, and use their Relative standard deviations (RSD%) of <40%. The validity of the assay and the integrity of the results are based on the Metabolomics Quality Assurance and Quality Control Consortium (mQACC) recommendations. For instance, we used a system suitability solution (SSS) for system stability and performance quality, an internal standard solution to control the sample variation, and pooled quality control samples to control the batch variations.

### 4.5. Data Processing

Chromatograms acquired with positive and negative ionization were processed separately with our in-house-developed software, NovaMT LipidScreener V.1 (Nova Medical Testing, Inc., Edmonton, AB, Canada). Nova Medical Testing, Inc. developed the Python-based proprietary software package for tailored use with The Metabolomics Innovation Centre’s (TMIC) Global (Untargeted) Lipidomics platform, a solution for untargeted lipidomics of biological samples that has been applied to more than 70 projects between 2020 and 2023 [58,59,60,61]. The package includes automated mass recalibration, peak picking, retention time correction using a set of deuterated standards added to all samples, data alignment (based on *m*/*z*, MS/MS fragmentation patterns, and retention time), data cleansing (handling of multiple adducts, in-source fragmentation, and contaminants), polarity merging, lipid annotations, quality control, batch effect correction, normalization based on a set of deuterated internal standards, and biostatistics. For this project, data processing was performed with a minimum intensity cut-off of 3000 cts, a signal-to-noise ratio threshold of 5, a minimum peak length of 6 spectra, a retention time tolerance of 4.0 s, a *m*/*z* tolerance of 5.0 mDa or 20.0 ppm, and filtering by detection in at least 80% of injections within one or more groups. Common contaminants, dimers, and multiple adducts of the same feature were removed during data processing. Features detected for positive and negative ionization were merged into a unique peak list using a neutral mass tolerance of 20 ppm and a retention time tolerance of 20.0 s, i.e., only the most intense feature was kept when ions were detected for positive and negative ionization within the tolerance limits.

The detected features were putatively annotated by a three-tier annotation approach based on tandem-MS spectral similarity (Tiers 1 and 2 annotations based on structural information) or mass-matches (Tier 3 annotations) using NovaMT LipidScreener (Nova Medical Testing, Inc., Edmonton, AB, Canada). First, features were annotated based on MS/MS spectral similarity in Tier 1 (MS/MS similarity score ≥ 500, precursor *m*/*z* tolerance of 20.0 ppm and 5.0 mDa, and fragment *m*/*z* tolerance of 25.0 ppm and 10.0 mDa) or Tier 2 (MS/MS similarity score < 500, precursor *m*/*z* tolerance of 20.0 ppm and 5.0 mDa, and fragment *m*/*z* tolerance of 25.0 ppm and 10.0 mDa). We performed MS/MS annotations with the MS-Dial LipidBlast MS/MS library, Bruker Human Metabolome Database (HMDB) Metabolite Library 2.0, and MassBank of North America [62] LC-MS/MS libraries (updated in August 2022) [63,64]. The employed MS/MS search procedure required matching at least 15% of fragments detected in the sample spectrum or the matched library to calculate similarity scores. For example, if twenty fragments were detected for a feature, a minimum of precursor plus three fragments had to match a library spectrum to calculate an MS/MS similarity score. A restriction of at least one matched fragment was applied for lipids with a small number of fragments (less than 7). Lipids that did not reach the minimum requirement of matched fragments were excluded. Unannotated features were mass-matched to a curated database of lipids in Tier 3 (*m*/*z* tolerance of 20.0 ppm and 5.0 mDa).

Lipids can have many isomeric forms with identical chemical formulas, masses, and MS/MS fragmentation patterns. The compounds may differ only in the position of double bonds, functional groups, or stereochemistry. Although powerful, the untargeted LC-MS/MS approach cannot distinguish these lipids. Definitive identification of lipids requires the determination of such factors and comparison to identical standards, which was not performed in this study. Multiple peaks are often annotated as the same lipid at the molecular species level (most annotations for Tiers 1 and 2) or the species level (Tier 3), corresponding to similar compounds with minor differences in their structures (positions of double bonds, positions of modifications, stereochemistry, etc.). A ten-tier filtering and scoring approach embedded in NovaMT LipidScreener was employed to calculate MS/MS match scores (Tiers 1 and 2), restrict the number of possible matches, and select the best identification for isomeric and isobaric species, *viz.*: MS/MS spectral similarity (Tiers 1 and 2), expected retention time range for each lipid class and length of fatty acyl residues, expected adducts for each lipid class, *m*/*z* error, carbon to double bond equivalent ratio, odd/even number of carbons in fatty acyl residues, presence of unexpected modifications, presence of vinyl-ether bonds (plasmalogens), expected method and sample sensitivity for each lipid subclass (including the expected presence of each lipid class in biological samples), and library source (e.g., libraries containing lipids previously found in biological samples versus computationally-generated libraries). Matches with unexpected retention times, adducts, and *m*/*z* errors above 5.0 mDa or 20.0 ppm were excluded.

Abbreviations for lipid classes and nomenclature followed the LipidMaps database (https://www.lipidmaps.org), the MS-DIAL LipidBlast spectral library (http://prime.psc.riken.jp/Metabolomics_Software/MS-DIAL/index5.html), and the Lipidomics Standard Initiative (https://lipidomics-standards-initiative.org, accessed on 25 July, 2023) (Supplementary Table S1). This work did not determine the position of double bonds or the stereochemistry of compounds. Tiers 1 and 2 annotations (MS/MS matches) were determined at the species or molecular species level, i.e., the definition of lipid classes, the composition of fatty acyl/alkyl residues (or summed composition if individual residues are not specified in the source database), and functional groups. When provided, common names were attributed based on biological intelligence, i.e., the most usual form of the molecule found in nature, rather than analytical evidence. Tier 3 annotations were defined at the species level, i.e., lipid class and subclass, total number of carbon atoms, double bond equivalents, and additional oxygen or other atoms [65,66].

A batch-effect correction was performed by linear interpolation of detected peak intensities with the QC aliquots injected immediately before and after the sample batch. We adopted a well-known approach for the normalization of lipidomics data to correct ion suppression and ion transmission effects, as well as small variations that may occur during sample handling, by using a mixture of 15 deuterated lipid standards that belonged to different lipid classes (NovaMT LipidRep Internal Standard Basic Mix for Serum/Plasma). The concentration of each standard was carefully optimized to ensure similar intensities for serum samples within the same retention time range for control of ion suppression. All annotated species were matched to the most similar internal standard based on lipid class similarity and retention time ranges. Intensity ratios were calculated, i.e., the intensity of the detected lipid was divided by the intensity of the most similar standard. The internal standard-normalized intensity ratios were further normalized to the median intensity within each sample and filtered by a relative standard deviation (RSD) smaller than 30% for QC replicates to remove features with low experimental reproducibility.

### 4.6. Statistical Analysis

Statistical analysis was performed using NovaMT LipidScreener and MetaboAnalyst 5.0 (http://metaboanalyst.ca). Only normalized peak intensity ratios for annotated compounds with an RSD smaller than 30% for QC replicates were employed. Non-informative features (i.e., internal standards, common contaminants, and features with low experimental reproducibility) were filtered out during data processing.

For univariate statistics, no extra filtering or data scaling methods were applied. Volcano plots (Mann–Whitney test for unequal variances, cut-off: FDR *p*-value ≤ 0.05, and FC 1.5) were used to select significantly altered lipids.

For multivariate statistics, features with near-constant values were filtered out (i.e., features related to homeostasis or unaffected by the studied conditions). The dataset were also auto-scaled and presented in principal component analysis (PCA) and partial least squares discriminant analysis (PLS-DA). OPLS-DA models were validated through 10-fold cross-validation by calculation of regression (R^2^, the explained variation, i.e., the “goodness of fit” between samples and their assigned groups) and prediction coefficients (Q^2^, the predictive relevance, i.e., a measure of the model’s ability to predict the group of new samples). Permutation tests were also performed to evaluate classification significance through *p*-values (100 permutations) [67].

Venn diagram analysis and a two-way ANOVA with an FDR corrected *p*-value (FDRp) cut-off = 0.05 were used to exclude the effect of confounding factors, including age and gender, on the overall data set. The gender- and age-independent lipid molecules were extracted sequentially after applying a two-way ANOVA analysis.

## 5. Conclusions

In summary, the current study provides new insight into our understanding of the lipidomics alterations associated with obesity, T2DM, and metformin. Also, it gives us a better understanding of the progression of diabetes from the perspective of lipid metabolism. Since the regulation of the annotated lipid molecules is disrupted, they could be used as biomarkers for assessing T2DM progression and treatment response, particularly after metformin administration. Lipidomic changes have yet to be widely implemented as an indicator of DM in clinical practice. Elevated body mass index (BMI), which is an obesity indicator, has been used as a clinical metric despite its limitations. There has been no current consensus regarding the use and application of lipid molecules as validated clinical prognostic or treatment response biomarkers of DM in the case of obesity. The significantly annotated dysregulated lipids as an effect of DM, obesity, and metformin use would be potential biomarkers. However, the findings of this study were based on single-center data, which might not sufficiently consider the multiple potential confounding factors such as ethnicity and region. Therefore, validating annotated potential lipid molecule biomarkers and their clinical utility are required in large-scale multicenter studies considering different cohorts.

## Figures and Tables

**Table 1:** Demographic data and the clinical characteristics of this study population.

Parameters	Non-Diabetic (*n* = 49)				Diabetic (*n* = 40)			
	Control (*n* = 28)		Obese (*n* = 21)		OT2DM (*n* = 16)	OT2DMMet (*n* = 24)		
	Mean	SEM	Mean	SEM	Mean	SEM	Mean	SEM
Age (Y)	26	11.5	34.9 *	22.4	48.7 *§	22.9	46.4 *§	22.2
Gender (M/F)	17/17 #	-	5/16 #	-	11/5 #	-	9/15 #	-
BMI (kg/m^2^)	23.1	2.9	38.7 *	17.5	33.6 *	14.9	39.7 §ǂ	14.3
FBG (mmol/L)	5.1	1	5.3	1	10.2 *§	9.7	9.6 *§	7.5
HbA1c (%)	5.6	0.5	5.2	3.4	8.4 *§	5.6	8.6 *§	3.9
LDL (mmol/L)	2.6	1.7	3.1	1.6	3.4 *	1.7	2.5 §ǂ	1.5
HDL (mmol/L)	1.4	0.4	1.2	0.5	1 *§	0.4	1 *§	0.4
TG (mmol/L)	0.8	0.6	1.1	1	2.2 *§	1.9	1.3 *ǂ	0.9

Abbreviations: OT2DM, obese type 2 diabetic; OT2DMMet, obese type 2 diabetic on metformin; SEM, standard error of the mean; BMI, body mass index; FBG, fasting blood glucose; HbA1c, glycated hemoglobin; LDL, low-density lipoprotein; HDL, high-density lipoprotein; TG, Triglycerides. Results are presented as Mean ± SEM.; * *p*-value < 0.05 vs. control subjects; § *p*-value < 0.05 vs. obese subjects; ǂ *p*-value < 0.05 vs. OT2DM (*p*-values were calculated based on the Mann–Whitney test.) # Chi square: (non-diabetic = 0.054, Diabetic = 0.052).

**Table 2:** Common and significantly altered lipid molecules as effects of BMI, T2DM, and metformin administration.

Lipid Molecules Characteristics					BMI Panel	T2DM Panel	Metformin Panel
Subclass	Level of Identification	Compound	RT (min)	Mass (*m*/*z*)	[Obese] vs. [Control]	([OT2DM] vs. [Obese])	[OT2DMMet] vs. [OT2DM]
					FC *	FC *	FC *
ST	Tier 3	ST 26:0;O4;H	1.5	602.4306	↑1.94 ***	↓0.31 ***	↑2.17 ***
MIPC	Tier 3	MIPC 32:4;O3	8.57	941.5348	↑1.95 ***	↓0.41 ***	↑1.74 *
PG	Tier 3	PG 40:5	9.58	847.548	↑3.10 **	↓0.29 **	↑4.74 *
TG	Tier 3	TG O-76:6	13.7	1196.091	↑3.13 **	↓0.30 *	↑4.76 *
	Tier 3	TG 52:4	18.8	872.7744	↑1.96 **	↓0.28 ***	↑2.31 *
PC	Tier 1	PC O-14:1_22:1	12.32	772.6243	↑2.49 *	↓0.30 **	↑4.82 *

Abbreviations: RT, Retention time; OT2DM, obese type 2 diabetic; OT2DMMet, obese type 2 diabetic on metformin; BMI, body mass index; ST, Sterol Lipids; MIPC, Mannosyl-inositolphosphoryl-ceramides; PG, Phosphatidylglycerols; TG, Triacylglycerols; PC, Phosphatidylcholines; FC, Fold change; ↑: up-regulated or ↓: down-regulated. * *p*-value < 0.05. ** *p*-value < 0.005, *** *p*-value < 0.00005.

## Data Availability

Data are contained within the article and Supplementary Materials.

## Supplementary Materials

The following supporting information can be downloaded at: https://www.mdpi.com/article/10.3390/ph16121717/s1. Table S1: Abbreviations employed for lipid classes; Table S2: List of identified and normalized features from in-depth global lipidomics analysis employed for statistics; Table S3: List of gender- and age-independent lipid molecules; Table S4: Lipidomic profile of obese versus lean subjects; Table S5: Lipidomic profile of obese diabetic versus obese non-diabetic; and Table S6: Lipidomic profile of obese diabetic with metformin versus obese diabetic.
